# The Nuclear Pore Complex and mRNA Export in Cancer

**DOI:** 10.3390/cancers13010042

**Published:** 2020-12-25

**Authors:** Katherine L. B. Borden

**Affiliations:** Department of Pathology and Cell Biology, Institute of Research in Immunology and Cancer (IRIC), Université de Montréal, Pavillion Marcelle-Coutu, Chemin Polytechnique, Montreal, QC H3T 1J4, Canada; katherine.borden@umontreal.ca

**Keywords:** RNA export, nuclear pore complex, CRM1, NXF1/NXT1, eIF4E

## Abstract

**Simple Summary:**

There are multiple processes that can go awry to drive cancer. One of these arises from a dysregulation of trafficking of cellular materials between the two major compartments of the cell—the nucleus and the cytoplasm. These compartments are separated by a membrane or “wall”, but in this wall reside a series of tunnels, or pores, that permit specific materials to transit. One of these materials, known as RNA, carries the information from the nucleus to the cytoplasm to make proteins that can act in certain cellular processes such as growth or survival. If these RNAs transit between compartments inappropriately, they can cause dysregulation of a wide array of cellular processes, which in turn can contribute to cancer. This review describes the relevant pathways and presents strategies to target this process in cancer.

**Abstract:**

Export of mRNAs from the nucleus to the cytoplasm is a key regulatory step in the expression of proteins. mRNAs are transported through the nuclear pore complex (NPC). Export of mRNAs responds to a variety of cellular stimuli and stresses. Revelations of the specific effects elicited by NPC components and associated co-factors provides a molecular basis for the export of selected RNAs, independent of bulk mRNA export. Aberrant RNA export has been observed in primary human cancer specimens. These cargo RNAs encode factors involved in nearly all facets of malignancy. Indeed, the NPC components involved in RNA export as well as the RNA export machinery can be found to be dysregulated, mutated, or impacted by chromosomal translocations in cancer. The basic mechanisms associated with RNA export with relation to export machinery and relevant NPC components are described. Therapeutic strategies targeting this machinery in clinical trials is also discussed. These findings firmly position RNA export as a targetable feature of cancer along with transcription and translation.

## 1. Overview

Dysregulation of many cellular processes contributes to cancer. Aberrant transcription, translation, and signaling are amongst the most widely studied. However, the entire journey of coding RNA from transcription in the nucleus to translation into protein in the cytoplasm is marked by important RNA processing steps such as m^7^G capping, splicing, polyadenylation, and subsequent export to the cytoplasm [1,2,3,4,5,6,7]. Indeed, the transcriptome does not always predict the proteome [8]; this arises in large part because of regulation at the post-transcriptional level, which can decouple transcription and translation. Not surprisingly, many aspects of RNA processing including RNA export are now known to be dysregulated in, and contribute to, cancer [1,2,3,4,5,6,7]. To be exported, RNAs associate with a wide array of adaptor proteins and are exported as large ribonucleotide complexes (mRNPs), which enable them to traverse the channel in the nuclear envelope known as the nuclear pore complex (NPC) [4,6,7]. Indeed, RNA export factors and NPC components regulate the export of selected RNAs that act in nearly all facets of malignancy, e.g., survival, proliferation, metastases, and invasion [4,6,7]. Many signaling pathways converge on the RNA export machinery as well as the NPC, thereby positioning this process as a mediator of signaling and stress responses [4,6,7]. In this review, we focus on mRNA export and the architectural features of the vertebrate NPC relevant to this activity.

## 2. General Features of Nuclear-Cytoplasmic Trafficking

**Figure 1:**
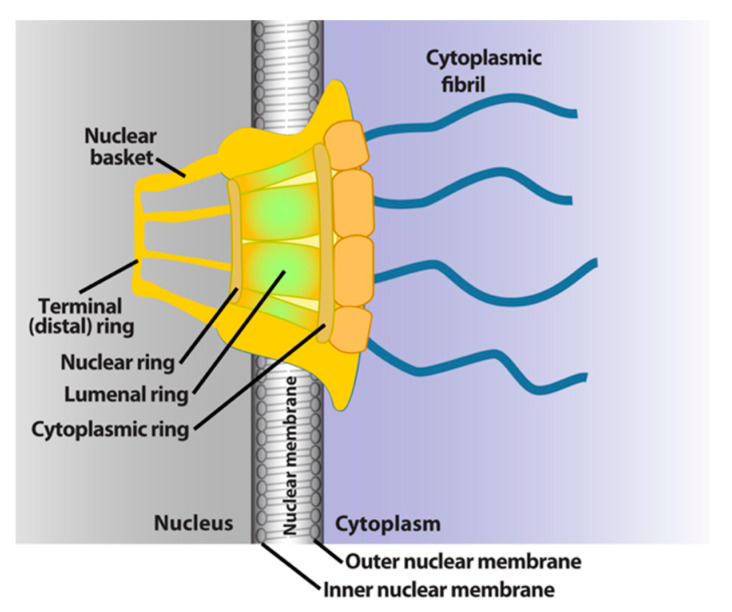
Schematics of the basic elements of the vertebrate nuclear pore complex. The nuclear basket, central pore, and cytoplasmic fibrils are shown. For simplicity, only four cytoplasmic fibrils and four nuclear basket protrusions are shown, rather than all eight.

The NPC is a megadalton protein complex embedded within the nuclear membrane and serves as the primary transit route between the nucleus and cytoplasm. This route is used by a wide array of macromolecules including mRNA. The structure of the NPC was first described in early EM studies as an apparently hollow cylindrical moiety embedded in the nuclear membrane with an eightfold symmetry around the pore [9,10]. The NPC has been the subject of intense structural studies and its basic structural features are conserved from fungi to humans [11,12,13,14] (and references therein). High-resolution cryo-electron tomography has been used to generate 3D reconstructions of intact NPCs while NMR and X-ray crystallography have been employed to elucidate the structures of subcomplexes at atomic resolution [11,12,13]. These studies reveal that the NPC is composed of three main structural features: a nuclear basket, a central membrane-traversing channel, and cytoplasmic fibrils (Figure 1). The nuclear basket and cytoplasmic fibrils are attached to the central framework of the pore through nuclear rings and cytoplasmic rings, respectively. The nuclear basket is formed by eight filaments joined by a distal ring, while eight cytoplasmic filaments or fibrils are anchored by the cytoplasmic rings (for clarity of presentation, only four are shown in Figure 1). In humans, the NPC has a molecular mass of ~10 MDa and is comprised of ~30 nucleoporins (Nups), which are present in multiple copies. The protein constituent Nups are attributed names on the basis of their molecular mass. The NPC is characterized with outer and inner diameters of approximately 120 nm and 40 nm, respectively [15,16]. The nuclear basket serves as a docking site for export cargoes with the NPC. The central channel is set within the nuclear envelope and is not hollow. Within the channel, intrinsically disordered phenylalanine–glycine (FG) repeat proteins constitute a diffusion barrier. Macromolecule cargoes smaller than ~30–50 kDa can traverse passively through the diffusion barrier whereas larger molecules require nuclear transport factors (also known as karyopherins) for successful translocation [11,17,18]. Characteristics of cargoes such as their surface charge can dramatically alter their permeability, allowing larger factors to diffuse passively in some instances [19,20,21]. On the cytoplasmic side, filaments known as cytoplasmic fibrils can project up to 50 nm outward into the cytoplasm [11,12,13]. These function as cargo release sites for export cargoes or docking sites for nuclear import [4,22,23,24]. It is important to note that NPC composition can differ in specific cell types, revealing context-dependent transport functions [25,26,27]. Further, the NPC and some associated factors can also act in non-transport functions [25,26,27,28]; however, we will focus on transport activities here.

Most cargoes must associate with nuclear transport receptors and associated factors to transit through the NPC. Cargoes typically interact with nuclear transport receptors such as importins, exportins, or transportins. For import of cargoes into the nucleus, transport typically requires protein cargoes to contain an accessible nuclear localization signals (NLS) [29]. In the cytoplasm, importins associate with cargoes displaying accessible NLSs and allow these to transit through the NPC to the nucleus. Once in the nucleus, cargoes must be released from the importin. This occurs via association of RanGTP with the importin, which induces conformational changes that lead to cargo release. Subsequently the RanGTP–importin complex is recycled to the cytoplasm [29,30,31]. In the cytoplasm, the RanGTPase-activating protein (RanGAP) in the presence of Ran-binding proteins (RanBPs) substantially stimulates hydrolysis of RanGTP, and the subsequent RanGDP dissociates from the cargo for release into the cytoplasm, freeing the importin for future rounds of nuclear import [29].

Export from the nucleus generally necessitates cargoes to possess an accessible nuclear export signal (NES), permitting association with exportin proteins. The prevalent exportin is known as exportin 1 or chromosome maintenance protein 1 (XPO1/CRM1). Here, cargoes with an accessible NES form a complex with an exportin and RanGTP [32,33,34]. This complex can now associate with factors at the nuclear basket and transit through the central channel of the nuclear pore. At the cytoplasmic side, one of the major components of the fibrils is Nup358, also known as RanBP2 [11,12,13]. RanBP2 contains docking sites for many proteins and binds RanGAP as well as binding sites for both RanGDP and RanGTP [12,13,24,35]. Here, the cargoes are released from the exportin by hydrolysis of RanGTP to RanGDP through RanGAP associated with RanBP2 or in some cases with the small, soluble RanBP1 [36]. Hydrolysis to RanGDP reduces the affinity of the exportin for the NES-containing cargo, allowing its release into the cytoplasm and subsequent recycling of the exportin to the nucleus for future rounds of export [37]. To ensure directionality of transport, Ran is recycled by the nuclear transport factor 2 (NTF2), which binds RanGDP in the cytoplasm and ferries it to the nucleoplasm [36]. Here, the RanGEF exchange factor RCC1 facilitates nucleotide exchange to regenerate RanGTP [36].

## 3. General Features of mRNA Export

mRNA export is extremely important for gene regulation, serving two critical functions: (1) it provides a surveillance mechanism, meaning that it weeds out aberrant mRNAs, and thus these do not become translated into aberrant proteins with altered functions. (2) It serves as an important regulatory step, permitting altered flow of specific mRNAs into the cytoplasm in order to control their translation into protein and thus the response to extracellular signals as well as stress conditions. Indeed, groups of mRNAs encoding proteins acting in the same biochemical pathways can be coordinately exported, or retained in the nucleus, providing a powerful means to quickly turn on or off biochemical pathways that drive cell physiology [38,39,40,41]. This model of how to control groups of mRNAs is referred to as RNA regulons. This coordinated control typically arises from the presence of cis-acting elements conserved amongst mRNA targets, which are referred to as USER (untranslated sequence elements for regulation) codes [38,39,40,41]. Transit through the NPC is by far the major exit route for export, but there are examples of large mRNPs exiting the nucleus by budding at the nuclear membrane [42]. In this review, the focus is on the NPC route. As will be described below, the NPC plays an active role in RNA export and indeed its modulation can impact on bulk mRNA export or on selected mRNAs.

**Figure 2:**
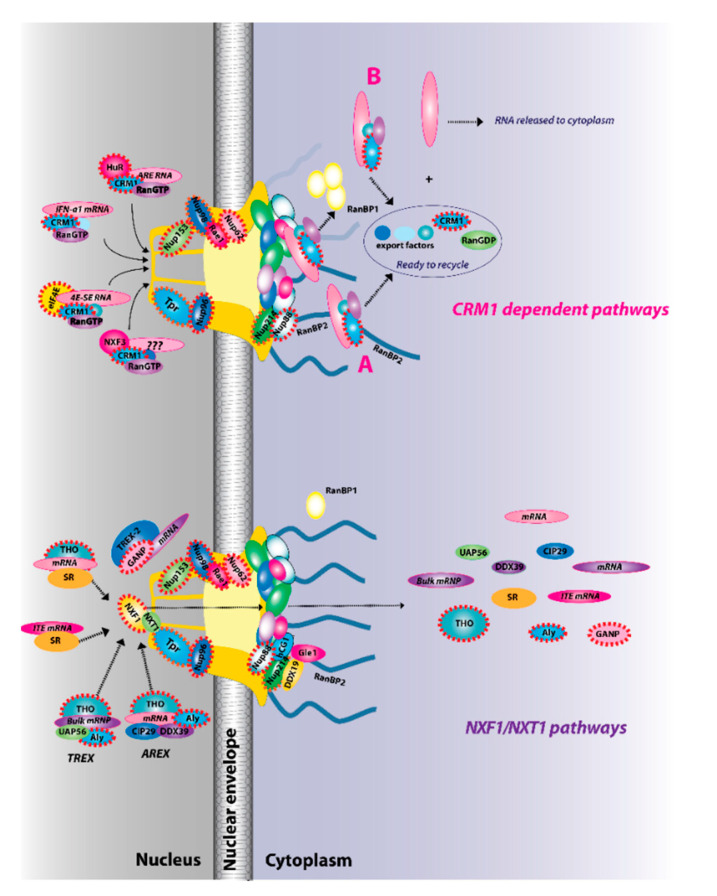
The two major RNA export receptor pathways, nuclear RNA export factor 1 (NXF1)/nuclear transport factor 2-like export 1 (NXT1) and chromosome maintenance protein 1 (CRM1), are shown, and both are dysregulated in cancer. There are multiple routes to engage either of these pathways. For example, for the NXF1/NXT1 pathways, there are complexes that rely on ALY/REF (transcription export (TREX) and AREX) and also on GANP (TREX2). There are the intronless *H2a* RNAs with intronless transport elements (ITEs) that rely on SR proteins but additionally some intron-containing RNAs also require SR proteins. For the NXF1/NXT1 pathway, cargo release depends on DDX9/Gle1. There are also multiple routes to engage the CRM1 pathways. These are depicted for *IFN1α*, HuR, eIF4E, and NXF3. For the CRM1 pathway, cargo release involves RanGTP hydrolysis through either the RanBP1 or RanBP2 pathways (**A**). For the case of eIF4E overexpression (**B**), RanBP2 levels are reduced (depicted by fibrils in shadow) and RanBP1 levels are increased. Thus, the RanBP1 release pathways are thought to predominate. Once cargoes are released from both the NXF1/NXT1 and CRM1 pathways, exportins and associated factors are recycled (not shown). Only four cytoplasmic fibrils and four nuclear basket protrusions are displayed for simplicity of presentation (as there are eight fibrils per nuclear pore complex (NPC)). Factors known to be involved in cancer are encapsulated in red dashed lines. This is not an exhaustive list of all NPC factors that are dysregulated in cancer but highlights the fact that all facets of the NPC can be impacted (nuclear basket, central channel, and cytoplasmic fibrils).

Given the fundamental roles mRNA export plays in gene expression, it is not surprising that RNA export is typically closely tied to RNA processing. For mRNAs, this generally involves the addition of a 7-methylguanosine (m^7^G cap) on the 5′ end of transcripts, splicing and addition of a polyA tail on the 3′ end of transcripts. Interactions with particular protein factors mark the mRNA as processed and ready for export [4,6]. Generally speaking, mRNA cargoes cannot directly interact with proteins at the nuclear basket. Rather, their protein co-factors influence the ability of cargo mRNPs to form complexes with nuclear receptors, which in turn mediate their interaction with the nuclear basket and subsequent traversal of the NPC [4,6]. For the export of the majority of RNAs, the m^7^G cap plays a key role permitting interactions with the cap-binding protein complex (CBC) or in some cases with the eukaryotic translation initiation factor eIF4E as well as other factors [6]. To date, the best studied mRNA export receptors are the nuclear RNA export factor 1 (NXF1)/Tip-associating protein (TAP) acting in complex with NXT1 (nuclear transport factor 2-like export 1)/p15 [43,44] or XPO1/CRM1 [4,6] (Figure 2). The majority of mRNAs use the NXF1/NXT1 heterodimer route [4,45]. RNA export can be modulated at several stages, including assembly of mRNPs in the nucleus, association with the nuclear basket, RNA cargo release in the cytoplasm, and recycling of export factors back to the nucleus. This provides multiple steps that can respond to extracellular stimuli, stress, intracellular signals, or can be dysregulated in cancer.

## 4. NXF1/NXT1 mRNA Export Pathways

NXF1 requires interaction with the small NXT1 co-factor to effectively associate with components of the NPC and thus to act in RNA export. Indeed, depletion of NXT1 results in nuclear accumulation of poly(A) transcripts [43,44]. The NXF1/NXT1 pathway is characterized by several features including its use of the transcription export (TREX) complex. TREX consists of UAP56, ALY/REF (ALY), CIP29, and the multi-subunit THO complex, which is comprised of THOC1/Hpr1, hTho2, THOC5, THOC6, THOC7, and Tex1 [46,47,48]. ALY, through interactions with the THO complex, bridges interactions between the cargo mRNAs and NXF1/NXT1 [46,47,48]. TREX is typically recruited to the transcripts during splicing [49]. TREX associates with the 5′ end of RNA [50], while ALY appears to interact with the 3′ end of transcripts [51]. ALY’s nuclear export functions are regulated by inositol polyphosphate multi-kinase and its enzymatic product phosphatidylinositol(3,4,5)-triphosphate [52]. In this way, signaling is tightly linked to RNA export activity. Recently, it has been suggested that ALY recognizes 5-methylcytosine (m^5^C) in mRNAs, which has been postulated to play a role in the export of some transcripts [53]. Several of the TREX-associated proteins can directly bind mRNA to promote export. These include ALY and THOC5 [54], which are considered critical for recruitment of NXF1 to the complex promoting direct association between RNA and NXF1/NXT1 [54]. Even within the NXF1/NXT1 pathways, there is significant diversity including alternatives to the TREX complex, e.g., TREX2 and the alternative TREX (AREX) export complexes [4,6] (Figure 2). Common to all of these is the use of NXF1/NXT1 to associate with the NPC through interactions with NPC components that act in RNA export, Rae1 and Nup98, which then allow passage through the central channel [55,56,57]. 

As alluded to above, nuclear basket components and associated factors, e.g., TPR, Nup153, Nup50, Rae1, and Nup98, play important roles in mRNA export [7,14,58,59,60,61]. These factors can have specific or general effects on export. For example, TPR depletion leads to accumulation of polyA RNA in the nucleus in some cell types [62,63,64]. These activities can be cell- and context-specific. For instance, during neurogenesis, a neuron-specific transcript *STX1b* undergoes splicing, export, and translation, while in non-neuronal cells, these RNAs are not spliced correctly, leading to nuclear retention and degradation, which is TPR-dependent [65]. Additionally, the TPR protein is tethered to the NPC via Nup153 [66], although there is some disagreement if this is required [67] (and references therein). Disruption of the TPR–Nup153 interaction leads to the leakage of intron-containing RNAs into the cytoplasm, showcasing the mRNA surveillance activity of the nuclear basket [63]. The TREX2 component GANP requires TPR for association with the NPC [67]. Indeed, depletion of Tpr more closely mirrored loss of NXF1 or GANP than did depletion of other nuclear basket components Nup153 or Nup50, also highlighting that basket components can have differential impacts on mRNA export [67]. Cell cycle-driven changes to the NPC also contribute to changes in mRNA export. For instance, ubiquitination and subsequent degradation of Nup96 during M and G1 phase permit export of selected RNAs including those that encode regulators for the G1/S transition [68]. Nup96 heterozygote mice have specific mRNA export defects in immune cells leading to increased virus susceptibility [69]. Thus, while these are central components of the NPC, they selectively impact specific mRNAs.

Once the mRNP cargoes arrive at the cytoplasmic face, they generally interact with the cytoplasmic fibrils of the NPC (Figure 2). The cytoplasmic fibrils are the location of most cargo release and subsequent recycling of export factors. Cargo release and recycling are important stages of the export process, and thus are highly regulated. RanBP2, a major constituent of the fibrils, contains binding sites for many proteins including NXF1/NXT1, RanGAP, Ran, and CRM1 [22,70,71,72]. RanBP2 is linked to the central channel via Nup88 and Nup214 [22]. RanBP2 hypomorph mice do not have bulk mRNA export defects but rather display specific effects with elevation of the export of selected mRNAs, whereas knockout of RanBP2 leads to severe impairments in bulk mRNA export [23,73]. Interestingly, hypomorph mice develop spontaneous cancers relative to littermate controls [73]. Most mRNA cargoes are released into the cytoplasm by the ATP-dependent DEAD box helicase (DDX19) and its co-factor Gle1 [74,75,76]. This step requires a potent signaling molecule inositol-hexakisphosphate (InsP6), where Gle1–InsP6 complex stimulates the binding of DDX19 to cargo mRNA, triggering ATP hydrolysis and cargo release [74,75,76]. Thus, signaling pathways can influence RNA export. Many of the components described here such as RanBP2, Nup214, and Nup88 can act in other forms of RNA export as well, providing excellent examples of the general plasticity and modularity of the system.

A variant of the NXF1/NXT1 export pathway involves serine/arginine-rich SR proteins [77,78,79] (Figure 2). Initial studies into the role of SR proteins in mRNA export focused on intronless *H2a* mRNAs. Two SR proteins, SRp20 and 9G8, associate with a 22-nucleotide element in the *H2a* mRNA, referred to as the intronless transport element (ITE), and recruit the NXF1/NXT1 heterodimer to facilitate export [77]. Follow-up studies revealed that these factors are essential for the export of some spliced RNAs as well [79,80]. For spliced transcripts, some SR proteins, e.g., hyperphosphorylated 9G8, are recruited to the pre-mRNA and then are hypo-phosphorylated after splicing permitting association with NXF1/NXT1 [79]. After transit through the pore, SR proteins are re-phosphorylated, presumably allowing release of the cargo and facilitating recycling to the nucleus of NXF1/NXT1 [79]. More recent studies demonstrated that SR proteins are implicated in the export of > 1000 mRNAs in an NXF1/NXT1-dependent manner. SRSF3 and SRSF7 couple alternative splicing and alternative polyadenylation to NXF1/NXT1-mediated RNA export [78]. In this way, these SR proteins can promote or impair export of alternatively processed transcripts by recruiting NXF1/NXT1 to nearby regulatory sites [78]. In all, this suggests that there are at least two classes of adaptors for the NXF1/NXT1 pathways, ALY/REF and SR proteins [78]. This provides a means to selectively export (or retain) subsets of mRNAs using the same nuclear receptor NXF1/NXT1 but different adaptors [78,79].

## 5. CRM-Mediated mRNA Export

A smaller subset of RNAs transit the nuclear pore through the CRM1/XPO1 pathway (Figure 2). CRM1 plays multiple roles, as it is the major protein nuclear export receptor in the cell but also exports small nuclear RNAs (U snRNAs) [70,81] and some pre-microRNAs [82]. mRNAs shorter than 300 nucleotides in length also utilize a U snRNA-type strategy for exit [83]. CRM1 interacts with its cargoes using the NES found in many shuttling proteins [32,70]. In this way, CRM1 does not directly bind to mRNA but rather to adaptor proteins that mediate the interactions with transcripts [70]. In the nucleus, CRM1 associates with cargo in the presence of RanGTP [70]. Release in the cytoplasm requires interactions with RanGAP and either RanBP2 or the small, soluble RanBP1. This allows hydrolysis of GTP and release of the mRNA cargo. As with NXF1 pathways described above, Nup88, Nup214, and RanBP2 play critical roles in the release and recycling steps for CRM1-dependent export [70].

There are multiple variants of CRM1-dependent mRNA export (Figure 2). CRM1 exports mRNA cargoes with various cis-acting elements or USER-codes and different adaptor proteins. For example, some RNAs that contain AU-rich elements (AREs) in their 3′ untranslated region (UTR) undergo CRM1-mediated export via the co-factor HuR [84]. HuR directly binds the ARE elements in these RNAs. If the CRM1 inhibitor leptomycin B (LMB) is used, export of AU-rich (and some other RNAs), but not bulk RNA, is impaired [84]. Interestingly, HuD, an HuR family member specific to neurons, is associated with RNA and NXF1, indicating that HuR family members are not restricted to CRM1-dependent mRNA export [85]. Interferon-alpha-1 (*IFNa1*) transcripts are exported in a CRM1-dependent, HuR-independent manner, indicating that other adaptors exist for mRNAs to engage the CRM1 pathway [86]. An NXF family member known as NXF3 binds specific mRNAs but does not appear to bind the Nups of the nuclear basket. Instead NXF3 uses CRM1 to transit the NPC [87]. Presumably, there are specific USER code(s) that allow the recruitment of these selected mRNAs to this pathway, but these are yet to be identified.

CRM1 plays an essential role in mRNA export mediated by the eukaryotic translation initiation factor eIF4E [88,89,90,91] (Figure 2). While most focus on eIF4E is on its role in the cytoplasm, it also localizes to the nucleus [92]. eIF4E forms nuclear bodies in many organisms, e.g., yeast, *Drosophila*, *Xenopus*, mouse, and human [92,93,94,95]. Its best characterized nuclear role is in the export of specific RNAs, thereby increasing their cytoplasmic concentrations and thus providing better availability to the translation apparatus, and, in some cases, increasing their translational efficiency in the cytoplasm as well [93,96]. Consistent with its requirement for CRM1, LMB impairs eIF4E-dependent mRNA export, while knockdown of *NXF1/TAP1* has no effect [89]. Biochemically, eIF4E requires target RNAs to have a m^7^G cap. Additionally, eIF4E requires translation targets to have a complex 5′ untranslated region (UTR), while for RNA export targets it needs a ~50-nucleotide element in their 3′ UTR denoted an eIF*4E* sensitivity element (4ESE) [89,90,91,93,96]. In future, other RNA export elements may also come to light. Genome-wide analyses revealed that there are ~3000 RNAs that bind to eIF4E in the nucleus and thus are likely targets of eIF4E-dependent RNA export, with many of these mRNAs acting in pathways driving malignant transformation [88,89,97,98,99,100]. Some of these eIF4E-target transcripts alter the surface architecture of cells, imbuing migration, invasion, and metastatic capacity [101]. By contrast, housekeeping transcripts, e.g., *GAPDH*, are neither mRNA export nor translation targets of eIF4E [93,96].

Biochemical studies demonstrate the leucine-rich pentatricopeptide repeat C-terminus protein (LRPPRC) directly binds the 4ESE RNA element and eIF4E simultaneously [90,91] (Figure 2). Thus, eIF4E binds the 5′ m^7^G cap, while LRPPRC recognizes the 4ESE element in the 3′ UTR of cargo mRNAs. Furthermore, LRPPRC directly binds to CRM1 [91]. Thus, LRPPRC acts as an RNA export assembly platform [90], and it appears that the eIF4E-4ESE RNA–LRPPRC–CRM1 complex represents a minimal export complex [91]. These interactions are also observed in the nuclei of human cells [90]. In cells, the nuclear eIF4E export complexes also contains UAP56 and hnRNPA1, but not NXF1, CBC, or REF/ALY [90]. Thus, this pathway shares elements with the bulk NXF1/NXT1 export pathway but also uses specific factors to underpin its selectivity.

It is important to note that aside from its RNA export and translation activities, eIF4E also promotes the capping [102] and 3′ end processing [103] of a subset of transcripts. Thus, eIF4E appears to act as an m^7^G-cap chaperone, escorting RNAs through multiple RNA processing steps [104]. This cap-chaperone model provides a biochemical basis for eIF4E’s ability to act in these diverse processes. Further, it indicates that eIF4E has multiple nuclear activities, but how these drive RNA export remains to be investigated. Importantly, this means that eIF4E is poised to modulate mRNA export beyond forming the export complex described above and indeed may feed into the NXF1/NXT1 pathways through involvement in processing RNA substrates for that pathway. This possibility remains to be tested. Consistent with this idea, through its RNA export activity, eIF4E elevates production of Gle1 and DDX19 in human cells, suggesting it can increase cargo release in the NXF1/NXT1 pathway as well [105].

## 6. Changes in the NPC Associated with mRNA Export and Cancer

Given the critical role of mRNA export, and more generally trafficking, it is not surprising that both the NPC and the mRNA export machinery can be dysregulated in cancer [106,107,108] (Figure 2, see factors encapsulated with dashed red line). Dysregulation of these factors depends on the context specific landscape *i.e.* loss of some factors is observed in one type of cancer while their elevation is found in other malignancies. For example, THOC1 (component of the TREX complex) is reduced in skin and testes cancer specimens but yet is highly elevated in primary lung, ovarian, and colon cancer specimens [109,110]. In breast cancer, THOC1 levels are correlated with increased tumor size and metastases [111]. Indeed, in this context reduction in THOC1 levels correlates with inhibition of mRNA export, and it is likely that these mRNAs encode pro-survival and proliferative factors, and thus its decrease leads to subsequent reduction in the oncogenic phenotype [111]. The RNA export factor ALY is elevated in oral squamous cell carcinoma patient specimens [112]. Recent studies indicate that mutation of NXF1 in mice can lead to disruptions in hematopoiesis in a lineage-specific manner [113], and NXF1 has been found to be mutated in chronic lymphocytic leukemia (CLL) patients [114,115,116]. The germinal center associated protein (GANP), a component of the TREX2 complex, is highly elevated in several types of lymphomas [117]. While GANP is associated with bulk mRNA export [118,119], more recent studies have suggested that reduction of GANP in human cells impairs export of selected RNAs [120]. In either case, GANP elevation in lymphomas likely promotes inappropriate recruitment of cargo mRNPs to the nuclear basket in order to promote export and thus protein production. In addition, the nuclear basket protein Rae1 is elevated in breast cancer [121].

The CRM1 pathway is also implicated in cancer, including gliomas and cervical and pancreatic cancers, as well as in several hematological malignancies including multiple myeloma [122,123,124,125,126]. CRM1 mutations, which impact on nuclear-cytoplasmic trafficking, have been observed in cancer, specifically B cell malignancies [127]. Reduction in CRM1 levels and/or mutations in CRM1 in some cell types reduced proliferation, suggesting a causal link between specific RNA export and/or protein export and cancer [122,123,124,125,126,127]. Nup88 is associated with cytoplasmic fibrils, where it plays roles in cargo release. Nup88 is overexpressed in ovarian, breast, mesothelioma, colon, and prostate cancer patient specimens, and its overexpression is typically associated with advanced tumors [128,129,130]. Interestingly, in healthy cells, Nup88 relies on heterodimerization of Nup214 for its protein stability; however, Nup214 is not elevated in these malignancies, indicating that this relationship can be decoupled in cancer [129]. In all, the NPC and its associated receptors and co-factors can be altered in, and contribute to, cancer.

Chromosomal translocations have been identified for many Nups and nuclear pore-associated proteins in cancer [107,108]. Nup98 is involved in at least 14 translocations, mainly associated with hematological malignancies including myelodysplastic syndrome (MDS), acute myelogenous leukemia (AML), and chronic myelogenous leukemia (CML) [107]. Nup214 translocations are present in rare forms of AML and acute non-lymphoblastic leukemias [107]. The nuclear basket protein TPR is also found in translocations [107]. TPR–Tkr1 translocation is associated with papillary thyroid cancers [131]. TPR–Met fusions, where Met is a receptor tyrosine kinase that controls morphogenesis, proliferation, survival, and migration, are found in gastric carcinomas [107,132]. Typically, the functions of the fusion protein are not related to transport and could be the driving feature in terms of cancer. However, in the case of RanBP2–ALK fusions, the fusion protein associates with the NPC [133] and thus could potentially modify functions there; however, this remains to be examined.

The inability to properly control the number of NPCs is also linked to tumorigenesis [134,135]. Indeed, the number of NPCs can vary between different cell types by orders of magnitude, and this is not simply a function of available nuclear envelope surface area as the NPC density also changes [136,137]. NPC numbers also change as a function of normal physiological processes such as differentiation [137,138]. The nuclear basket protein TPR negatively regulates NPC numbers in human cells [139]. This activity is controlled via the ERK signaling pathway [139]. This provides a means to link proliferative signaling and NPC number and thus could be related to cancer.

The first example that a single protein could reprogram the NPC comes from eIF4E and relates to the major cytoplasmic fibril protein RanBP2 [105]. This reprogramming is linked to eIF4E’s oncogenic activity. eIF4E is highly elevated in a broad array of human cancers where this typically correlates with poor prognosis [45]. eIF4E overexpression leads to tumor formation in mouse models and to oncogenic transformation in immortalized cell lines [140,141,142,143,144]. Previous mutational studies indicated that the mRNA export activity of eIF4E substantially contributes to its oncogenic functions by promoting the expression of target mRNAs that encode proteins acting in nearly all facets of malignancy [93,101,105,145]. eIF4E overexpression leads to downregulation of RanBP2 and a partial relocation of Nup214 from the NPC to the nucleoplasm [105]. RanBP2 reduction leads to enhanced eIF4E-dependent mRNA export with no effect on the bulk mRNA export pathway [105]. Conversely, overexpression of a RanBP2 fragment that binds CRM1 impairs eIF4E-dependent mRNA export, presumably by sequestering CRM1 [105]. eIF4E overexpression causes a loss of contact inhibition [93,105], one of the hallmarks of cancer. eIF4E’s mRNA export activity is directly related to this effect [93,105]. Consistently, RanBP2 overexpression suppresses the ability of eIF4E to form foci [105]. As mentioned above, RanBP2 hypomorph mice develop more spontaneous tumors than littermate controls [73]. This suggests that the loss of control of eIF4E-mediated mRNA export could contribute to the oncogenic phenotype. Importantly, RanBP2 can also function in mitosis, and this likely also impacts its oncogenic activities [73].

Given the central role that the RanBP2 fibrils play in cargo release and recycling [70], it is clear that eIF4E must introduce a compensatory mechanism in order to permit multiple rounds of export. Indeed, the soluble co-factor RanBP1 is a direct mRNA export target of eIF4E, and consistently, eIF4E overexpression leads to increased RanBP1 levels [105]. RanBP1 is only 25 kDa and soluble. In this model, RanBP1 enables mRNA cargo release and CRM1 recycling more efficiently than with RanBP2, where RanBP2 can be associated with slower release due to sequestration on the large cytoplasmic fibrils [105]. These findings demonstrated that the NPC could be reprogrammed by oncogenes and related factors. In this case, this reprogramming is poised to impact many target mRNAs [97].

## 7. Therapeutic Targeting of mRNA Export in Cancer

eIF4E levels are increased in many cancers, where it generally correlates with poor prognosis [96]. In a subset of AML patients, eIF4E is substantially elevated and forms abnormally large nuclear bodies relative to early progenitor CD34+ cells or bone marrow mononuclear cells from healthy volunteers [145,146,147,148]. High-eIF4E AML is found in French American British (FAB) M4/M5 AML subtypes as well as a substantial portion of M1 and M2 AML subtypes (>150 specimens examined to date) [145,146,147,148]. While FAB subtypes are no longer used to classify AML, this showcases that a substantial number of AML patients have elevated, nuclear eIF4E relative to healthy volunteers. The nuclear enrichment of eIF4E in these AML specimens correlates with elevated eIF4E-dependent mRNA export relative to normal cells [145,146,147,148]. The contributions of its mRNA export activity to its oncogenic phenotype have been observed in several cancers, e.g., AML, diffuse large B cell lymphoma (DLBCL), and infant acute lymphoblastic leukemia [97,100,146,147,149].

eIF4E appears to play causative roles in malignancy given its overexpression promotes foci formation, growth in soft agar, and apoptotic rescue from a variety of stimuli [45,96,101]. In xenograft mouse models, elevated eIF4E correlates with increased tumor numbers, invasion, and metastases [143]. In transgenic models of eIF4E overexpression, mice develop a variety of cancers [140]. eIF4E-mediated transformation was thought to rely only on increased translation of oncogenic mRNAs [98]. However, eIF4E’s mRNA export functions are also critical for its oncogenic activities, as shown by mutational studies dissecting the role of translation and export and their relative impact on cancer [88,89,93,105,150,151]. eIF4E’s nuclear import via importin 8 and ability to modify the nuclear pore are also central to its oncogenic activity [105,149]. Indeed, addition of an NLS to eIF4E is sufficient to enhance its oncogenic activities, presumably by increasing eIF4E’s recycling to the nucleus after each round of export [91,149].

eIF4E has been targeted with multiple strategies in clinical trials. To date, the most promising studies have involved ribavirin, an old antiviral drug, which acts as a m^7^G-cap competitor, directly binding eIF4E as shown by NMR and other biophysical techniques [152,153,154]. Ribavirin inhibits eIF4E’s activities in mRNA export, translation, and oncogenic transformation [148,152,153,155,156]. RNAi knockdown of eIF4E reduces ribavirin activity, supporting it acts via eIF4E [157,158]. This prompted three clinical trials targeting eIF4E in AML patients, which resulted in objective clinical responses including remissions [146,147] (as well as ClinialTrials.gov NCT02073838). In the ribavirin monotherapy trial, there were 6/15 objective responses, including 1 complete remission (CR), 2 partial remissions (PR), 3 blast responses ((BR) where a blast response is defined as a drop in 50% or more of the leukemic blasts), and 6 stable diseases (SD) [146]. In the second trial, the combination of low-dose AraC with ribavirin, there were 5/14 objective responses for those patients who had over 20 uM ribavirin plasma levels including 2 CR, 1 PR, 2 BR, and 2 SD [147]. In patients, ribavirin blocks eIF4E’s association with importin 8, leading to cytoplasmic retention of eIF4E, impaired eIF4E-dependent mRNA export, and clinical responses [146,147,149]. Relapse correlated with nuclear re-entry of eIF4E and increased mRNA export due to chemical deactivation of ribavirin [146,149,152]. Other groups also completed early stage clinical trials targeting eIF4E with ribavirin, e.g., castration-resistant prostate cancer and head and neck cancers, and observed objective clinical responses [159,160]. There are >15 ongoing trials using ribavirin to target eIF4E in cancer (see https://clinicaltrials.gov as of 24 December 2020).

Other efforts have been made to target eIF4E in cancer. In mouse models, eIF4E was targeted with an antisense oligonucleotide (ASO) with promising results in a prostate cancer mouse model [161]. Unfortunately, in humans, the eIF4E ASO strategy was not as effective at reducing eIF4E levels and there were no objective clinical responses in 15 patients examined beyond stable disease for 7 patients and with no patient on trial for more than 3 months [162]. Similarly, targeting eIF4E indirectly via mTOR with a rapamycin analogue yielded 1 patient with a hematological improvement out of 22 AML patients [163]. Given the multiple roles of eIF4E, it is clear that targeting mRNA export is related to disease burden, but other facets of eIF4E activity likely also correlate with its oncogenic activities, and ASO, rapamycin, and ribavirin would target all of its cap-dependent activities.

## 8. Targeting CRM1 in Cancer

There has been substantial interest in targeting CRM1 in patients given its multiple roles in export. For example, inhibition of CRM1 represses eIF4E-mediated mRNA export [89,90,91]. CRM1 is also involved in export of other RNAs via HuR, as described above, as well as protein export, and thus all of these activities likely contribute to its clinical impact. Depletion of CRM1 or its pharmacological inhibition restored drug sensitivity towards many chemotherapies such as doxorubicin, etoposide, and others in cell lines [164]. The first identified CRM1 inhibitor was LMB [165]. LMB forms a covalent bond with Cys 528 of CRM1 in the same groove used to bind to NES signals on protein cargoes [32,166]. In this case, the lactone ring of LMB is hydrolyzed. After hydrolysis, LMB forms additional interactions with CRM1. In the absence of the lactone ring, the LMB derivative slowly de-conjugates, which is not observed with the parent LMB compound [166]. In early phase clinical trials in cancer carried out prior to the molecular understanding of its activity [167], LMB treatment resulted in overt toxicity, even at low doses, and there was found to be no clinical benefit, leading to a halt in these studies [167]. Consistent with this observation, LMB irreversibly blocks export in both cancer and in many normal cells, which leaves little in the way of a therapeutic window [168].

Despite these early findings, next-generation CRM1 inhibitors known as selective inhibitors of nuclear export (SINE) have shown some clinical success. SINEs also form covalent bonds with Cys 528 of CRM1, but this bond is slowly reversible, likely due to the absence of a lactone group, thereby preventing hydrolysis [168]. Indeed, SINEs such as KPT-330 (selinexor) interact with CRM1 in a slowly reversible manner [125,168]. Additionally, SINEs induce CRM1 degradation with subsequent re-synthesis, and thus CRM1 activity is not permanently blocked in these cells [125,166,169]. In all, these features of SINEs likely account for reduced toxicity in patients relative to LMB. Selinexor has been tested in many types of cancers, with promising results in some [168]. To date, it is approved by the US Food and Drug Administration for the treatment of relapsed or refractory multiple myeloma [170,171,172].

## 9. Other Disorders Involving NPC and RNA Export

There are several other disorders associated with dysregulated NPC components, some of which directly impact on mRNA export [26]. Mutations in Gle1 are associated with two genetic disorders: LCCS1 (lethal congenital contracture syndrome 1) and LAAHD (lethal arthrogryposis with anterior horn cell disease) [173]. Here, mutations lead to defects in Gle1-mediated cargo release from the NPC [173,174]. Gle1 also acts in translation [175], and thus these other functions may also contribute to the phenotypes observed. However, Gle1 oligomerization is perturbed by the disease-causing mutations, and this property is required for its mRNA export but not its translation activity, strongly suggesting that it is disturbed mRNA export that contributes to these disorders [174]. Mutation in Nup155 leads to cardiac disorders where Nup155 is in the inner ring and associated with Gle1. Mutations in Nup155 associated with disease by altering Nup155 localization and NPC permeability, leading to reduced *HSP70* mRNA export among other aberrancies [176,177]. It has been suggested that disruption of Gle1-dependent mRNA export causes atrial fibrillation [178], but this has yet to be established. The NPC and mRNA export can also be impacted by viral infections. One example comes from vesicular stomatitis virus (VSV) work, which show that the VSV M protein promotes the export of specific viral RNAs. Here, the VSV matrix M protein disrupts interactions on the nuclear basket between Nup98 and Rae1 [56]. mRNA export is also disrupted in several neurodegenerative conditions [179]. Furthermore, NPCs are known to change composition upon oxidative stress [68,180]. Thus, modulation of NPC components and mRNA export can impact on a wide array of events, ultimately impacting on cell physiology.

## 10. Conclusions

Regulation of mRNA export provides a mechanism to rapidly control the proteome without necessitating further transcription. The multiplicity of exit strategies for RNAs affords an elegant molecular basis for selectively. Aberrant mRNA export can lead to dysregulation of a wide variety of processes that support malignancy. The ability of mRNA export to respond to signaling implicates it as a key integrator of gene expression and cell physiology. mRNA export machinery and relevant NPC components are dysregulated in a variety of human cancers. Recent clinical studies suggest that targeting these pathways could lead to clinical benefit. Importantly, many of the factors described here also play roles in addition to mRNA export, and thus their other activities likely also contribute to the observed phenotypes. In all, mRNA export is an important step in gene regulation, contributes to a diverse set of human malignancies and can be targeted in patients, which in some cases is associated with clinical benefit.
